# Isolated ovarian tuberculosis in a hemodialysis patient: An incidental pre-transplant discovery with diagnostic and management implications

**DOI:** 10.1016/j.idcr.2026.e02551

**Published:** 2026-03-17

**Authors:** Ayyoub Hormatallah, Ikram Asakak, Loubna Slama, Mahmoud Aberkane, Mohamed Harmouche, Zainab Chatbi, Ibtissam Bellajdel, Hafsa Taheri, Hanane Saadi, Ahmed Mimouni

**Affiliations:** Mohammed I University Oujda Faculty of Medicine and Pharmacy Oujda, Oujda, Morocco

**Keywords:** Ovarian tuberculosis, Kidney transplantation, Immunocompromised host, Hemodialysis, Adnexal mass, Pre-transplant screening

## Abstract

Female genital tuberculosis remains a rare extrapulmonary manifestation, particularly when presenting as isolated ovarian involvement mimicking malignancy. We report a case discovered incidentally during pre-transplant screening in an immunocompromised patient on hemodialysis. A 37-year-old woman with end-stage renal disease on hemodialysis for 5 years underwent routine pre-kidney transplant evaluation. Computed tomography revealed a multiloculated left adnexal mass (38 × 33 mm). She was asymptomatic without tuberculosis exposure history. Transvaginal ultrasound demonstrated a thin-septated avascular cystic lesion, while magnetic resonance imaging showed hyperintensity with fluid-fluid levels and no suspicious enhancement. Laboratory findings revealed mild anemia, elevated inflammatory markers, and mildly elevated cancer antigen 125 with normal human epididymis protein 4. Tuberculosis screening showed positive tuberculin skin test (12 mm) and positive interferon-gamma release assay, with normal chest radiography. Laparoscopic cystectomy was performed. Histopathology demonstrated epithelioid granulomas with Langhans giant cells, caseous necrosis, and rare acid-fast bacilli. Molecular testing and culture confirmed Mycobacterium tuberculosis without rifampin resistance. Anti-tuberculous therapy adapted to renal failure was initiated with favorable outcome. At 6-month follow-up, complete resolution was documented on pelvic ultrasound. At 12 months, with no recurrence and normalized inflammatory markers, the patient was successfully relisted for kidney transplantation. This case emphasizes the importance of maintaining high clinical suspicion for tuberculosis in immunocompromised patients presenting with adnexal masses, particularly those awaiting transplantation. Early microbiological diagnosis enabled conservative surgical management and timely treatment, preventing potentially life-threatening post-transplant reactivation.

## Highlights

• Rare isolated ovarian TB in hemodialysis patient found pre-transplant.
• CA-125 elevation mimicked malignancy requiring surgical exploration.
• Laparoscopic diagnosis avoided extensive surgery and preserved fertility.
• Renal-adjusted TB therapy achieved cure before kidney transplantation.
• First case linking dialysis, ovarian TB, and transplant management.

## Introduction

Genitourinary tuberculosis is the second most common extrapulmonary site of tuberculosis after lymph node involvement [1]. In women, female genital tuberculosis most often affects the fallopian tubes and endometrium and is a recognized cause of tubal infertility in endemic settings [2]. It remains rare in industrialized countries and more frequent in endemic regions [3]. The true incidence is likely underestimated because diagnoses are often missed [4]. Clinical presentation is frequently nonspecific, with variable gynecologic symptoms or chronic pelvic pain, and approximately 11% of cases are asymptomatic and detected incidentally [5].

Ovarian involvement is particularly uncommon [6]. Although the ovaries may be involved in 20–30% of female genital tuberculosis cases [3], strictly isolated ovarian disease without tubal or uterine involvement is exceptional [7]. When present, this pattern can mimic ovarian cancer clinically and radiologically, with an adnexal mass, ascites, and sometimes elevated cancer antigen 125 (CA-125) [6]. Our case illustrates this pseudotumoral presentation in a 37-year-old woman with end-stage renal disease who was being evaluated for kidney transplantation, in whom ovarian tuberculosis was discovered incidentally during the pre-transplant workup. This highlights the need to include tuberculosis in the differential diagnosis of otherwise unexplained adnexal masses, even in the absence of specific symptoms or suggestive pulmonary disease.

Case Presentation

A 37-year-old gravida 3, para 1 woman with regular menses and end-stage renal disease secondary to chronic nephropathy of unknown origin for 7 years had been on three times weekly hemodialysis for 5 years via a left brachiocephalic arteriovenous fistula, without recent infectious complications. Human immunodeficiency virus, hepatitis B virus, and hepatitis C virus serologies were negative. She had no diabetes and did not smoke. She received Bacillus Calmette-Guérin vaccination in childhood and reported no personal or family history of tuberculosis and no known exposure. Surgical history included right adnexectomy for ectopic pregnancy 4 years earlier.

**Fig. 1:**
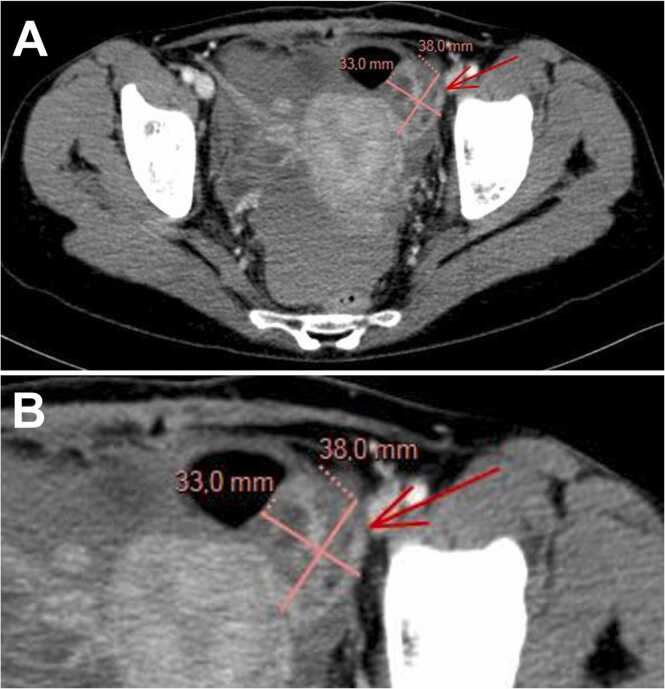
**Abdominopelvic computed tomography (axial soft-tissue window).** (A) Overview showing a multiloculated left latero-uterine adnexal mass (arrow). (B) Magnified view demonstrating thin septations with approximate diameter of 38 × 33 mm. No ascites and no visible pelvic lymphadenopathy.

A routine pre-transplant computed tomography scan of the chest, abdomen, and pelvis incidentally revealed a multiloculated left adnexal mass (Fig. 1). The patient had no gynecologic symptoms and no systemic signs. On examination, she was well-appearing: temperature 36.8°C, blood pressure 120/75 mmHg, pulse 80 beats per minute, and body mass index 21 kg/m². The abdomen was soft and non-tender without ascites; a remote Pfannenstiel scar was noted. Speculum and bimanual examinations were unremarkable; the breast examination was normal.

Laboratory testing showed hemoglobin 7.7 g/dL, lymphocyte count 600/μL, C-reactive protein 8 mg/L, erythrocyte sedimentation rate 35 mm/h, and albumin 34 g/L. Electrolytes were normal. Tumor markers showed CA-125 mildly elevated at 39 U/mL (normal <35), human epididymis protein 4 (HE4) 70 pmol/L (normal for premenopausal), alpha-fetoprotein 3.56 ng/mL (normal <10), and carcinoembryonic antigen 4.25 ng/mL (normal <5). Quantitative beta-human chorionic gonadotropin was negative.

**Fig. 2:**
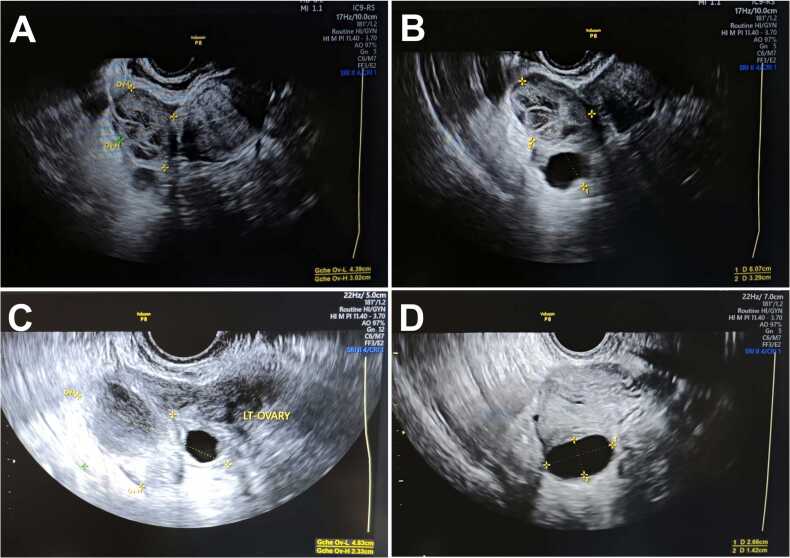
**Transvaginal ultrasound.** (A) Longitudinal B-mode view showing a multiloculated cystic mass (48 × 32 × 30 mm) in the left ovary. (B) Transverse view (zoom) with thin septations and multiple locules without vegetations. (C) Additional view confirming multiloculation with smooth walls. (D) Detail of an isolated locule with thin wall and homogeneous anechoic content.

**Fig. 3:**
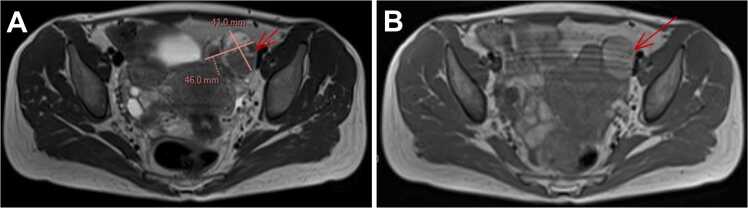
**Pelvic magnetic resonance imaging.** (A) Axial T2 sequence showing a multiloculated left ovarian cystic mass (41 × 46 mm) with a fluid-fluid level. (B) Axial T1 sequence showing intracystic hyperintensity without suspicious enhancement, consistent with proteinaceous or hemorrhagic content.

Transvaginal ultrasound demonstrated a left adnexal multiloculated cystic lesion measuring 48 × 32 × 30 mm, with thin septations, no Doppler flow (score 1, resistance index > 0.8), and no obvious solid component (Fig. 2). Pelvic magnetic resonance imaging showed a left ovarian cystic mass with T1/T2 hyperintensity and a fluid-fluid level, without suspicious enhancement or diffusion restriction, measuring 41 × 46 mm (Fig. 3). Across modalities, the measured size varied slightly (computed tomography ∼38 × 33 mm, ultrasound 48 × 32 × 30 mm, magnetic resonance imaging 41 × 46 mm), which is expected due to differences in technique, slice orientation, and cyst shape; all studies consistently supported a benign-appearing multiloculated cystic morphology.

Tuberculosis workup showed a normal chest X-ray. The tuberculin skin test measured 12 mm and the interferon-gamma release assay was positive. Three sputum samples were negative for acid-fast bacilli. Urine testing for mycobacteria was negative, arguing against concomitant urogenital involvement. No other extragenital focus was identified.

Differential diagnoses included endometrioma, tubo-ovarian abscess, and epithelial ovarian tumor. The absence of fever, normal tumor markers, lack of Doppler vascularity, and histologic proof helped exclude these entities. Because diagnostic uncertainty persisted, exploratory laparoscopy was performed for diagnostic and therapeutic purposes after informed consent, with the option of cystectomy or oophorectomy if malignancy was suspected. Adhesiolysis revealed an adhesive pelvis (parietal-intestinal, parietal-omental, and uterine-intestinal adhesions, score 2). The left ovary contained three cysts of about 2 cm each, filled with yellowish serous fluid, without ascites. Left cystectomy was performed with peritoneal biopsies and peritoneal washings sent for pathology and microbiology.

**Fig. 4:**
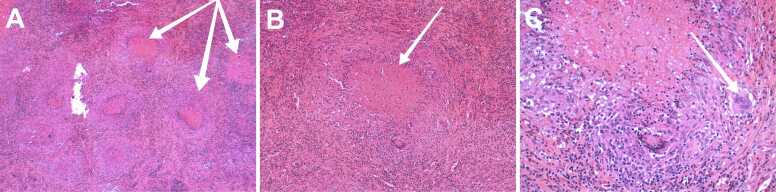
**Histopathology (hematoxylin-eosin-saffron).** (A) Overview at × 4 magnification with fibrous wall and multiple epithelioid granulomas. (B) Granuloma at × 10 magnification with central caseous necrosis. (C) Detail at × 20 magnification showing Langhans giant cells and peripheral epithelioid cells.

Histopathology showed thickened fibrous cyst walls and epithelioid granulomas with Langhans giant cells and caseous necrosis (Fig. 4). Ziehl-Neelsen staining revealed rare acid-fast bacilli; periodic acid-Schiff and Grocott stains were negative. No malignancy was identified. Tissue specimens were sent for mycobacterial culture on Löwenstein-Jensen medium, which confirmed Mycobacterium tuberculosis after 4 weeks with full drug susceptibility to isoniazid, rifampin, pyrazinamide, and ethambutol. Xpert MTB/RIF assay was positive for M. tuberculosis complex without rifampin resistance. A diagnosis of isolated ovarian tuberculosis was established.

Based on an estimated dry weight of 55 kg and a body mass index of 21 kg/m², anti-tuberculous therapy tailored to renal failure was initiated according to guidelines. The intensive phase consisted of 2 months of isoniazid 300 mg once daily, rifampin 600 mg once daily, pyrazinamide 1500 mg three times weekly administered post-hemodialysis, and ethambutol 800 mg three times weekly post-hemodialysis. The continuation phase consisted of 4 months of isoniazid 300 mg once daily and rifampin 600 mg once daily, with pyridoxine 25 mg daily throughout to prevent isoniazid-induced neuropathy. Tolerance was good and adherence was verified.

The course was favorable, with improved general condition and hematologic indices and normalization of C-reactive protein. A pelvic ultrasound at 6 months documented no residual adnexal lesion. Tumor markers remained within normal limits. At 12 months, no recurrence was observed. She was considered cured on clinical, laboratory, and imaging criteria, and she was actively relisted for kidney transplantation after completing therapy.

## Discussion

This case is one of the few reports of strictly isolated ovarian tuberculosis, and it is particularly notable because it was discovered incidentally in an asymptomatic patient during pre-transplant evaluation. The absence of symptoms aligns with prior data suggesting that female genital tuberculosis can remain unnoticed in about 11% of cases [5]. By contrast, our patient had important risk factors. End-stage renal disease on hemodialysis is an acquired immunodeficiency state associated with a markedly increased risk of tuberculosis, with reported incidences approximately 6–25 times higher than in the general population [8]. This background likely contributed to the subclinical course in this case. Importantly, diagnosing and treating tuberculosis before transplantation is advantageous, since post-transplant immunosuppression promotes reactivation of latent infection, most often within the first year, with tuberculosis incidences 3–15 times higher than in the general population [9]. This justifies systematic screening and, when latent infection is identified, preventive therapy [9].

The initial radiologic picture was that of an isolated cystic adnexal mass. Such a presentation poses a diagnostic dilemma with ovarian neoplasms, especially in the absence of a known tuberculosis context. Published data confirm that genital tuberculosis can closely mimic ovarian cancer clinically and on imaging: adnexal masses may appear heterogeneous on ultrasound or computed tomography, there may be ascites and lymphadenopathy [10], and CA-125 is frequently elevated in both conditions [6]. Several reports describe cases initially managed as ovarian tumors until histopathology revealed tuberculosis as the underlying etiology [11]. In our patient, the diagnosis was established on the resected ovarian specimen by identifying epithelioid granulomas with caseous necrosis and Langhans giant cells. For isolated extrapulmonary disease, histology or culture remains the diagnostic reference, and routine microbiology may be negative or not performed [4], [12]. When preoperative features suggest tuberculosis, image-guided or laparoscopic biopsy, with acid-fast staining and molecular testing, can secure the diagnosis and may allow conservative management before definitive surgery [4], [6], [13]. In the absence of suspicion, however, the diagnosis is often made only after surgery [11], [14].

Management of genital tuberculosis is primarily medical. A six-month multidrug regimen identical to that used for drug-susceptible pulmonary tuberculosis, namely two months of isoniazid, rifampin, pyrazinamide, and ethambutol followed by four months of isoniazid and rifampin, generally achieves cure [6], [15]. In renal failure, dosing must be adapted. Isoniazid and rifampin can usually be given at standard doses, pyrazinamide can be maintained at standard doses but scheduled after dialysis, and ethambutol should be reduced or given intermittently when creatinine clearance is below 30 mL per minute. Aminoglycosides should be avoided when possible [16]. Routine pyridoxine supplementation helps prevent isoniazid-induced neuropathy [15]. Outcomes are typically favorable under appropriate therapy [17]. In our case, timely identification allowed full anti-tuberculous treatment before immunosuppression, likely preventing severe post-transplant complications [9], [18].

From a reproductive standpoint, delayed diagnosis of genital tuberculosis can compromise ovarian function and fertility. Genital tuberculosis has been recognized as a major cause of tubal factor infertility, accounting for 44–74% of infertility in women with this condition in endemic regions [19]. Early recognition and medical treatment are therefore critical to preserve reproductive potential. More broadly, this case underscores that although ovarian involvement is uncommon, tuberculosis remains a great imitator in the differential diagnosis of adnexal masses [13], [20]. A high index of clinical suspicion is needed in at-risk or immunocompromised patients, and well-conducted anti-tuberculous therapy usually achieves cure while avoiding unnecessary extensive surgery [6].

## Conclusions

Tuberculosis remains a great imitator in the pelvis. In immunocompromised patients, particularly those on hemodialysis and candidates for kidney transplantation, a cystic adnexal mass with noncontributory tumor markers and equivocal imaging should prompt consideration of genital tuberculosis.

Early tissue diagnosis is essential. Targeted biopsy or limited excision with histopathology, acid-fast staining, mycobacterial culture, and Xpert Mycobacterium tuberculosis/rifampin can establish the diagnosis and help avoid extensive surgery. Management is primarily medical, with dose adjustments for renal failure and routine pyridoxine supplementation. Incorporating targeted tuberculosis screening into the pre-transplant evaluation can prevent post-transplant reactivation and safeguard the transplant pathway.

## CRediT authorship contribution statement

**Mahmoud Aberkane:** Writing – review & editing, Validation, Methodology, Formal analysis. **Ibtissam Bellajdel:** Writing – review & editing, Investigation, Data curation. **Hafsa Taheri:** Writing – review & editing, Investigation, Data curation. **Mohamed Harmouche:** Writing – review & editing, Validation, Methodology. **Zainab Chatbi:** Writing – review & editing, Investigation, Data curation. **Ayyoub Hormatallah:** Writing – review & editing, Writing – original draft, Project administration, Investigation, Conceptualization. **Hanane Saadi:** Writing – review & editing, Validation, Supervision. **Ahmed Mimouni:** Writing – review & editing, Validation, Supervision. **Ikram Asakak:** Writing – review & editing, Investigation, Data curation. **Loubna Slama:** Writing – review & editing, Investigation, Data curation.

## Ethical Approval

This case report was conducted in accordance with the ethical standards of the institutional research committee and with the 1964 Helsinki Declaration and its later amendments. Institutional review board approval was not required for this single case report as per institutional guidelines.

## Patient Consent

Written informed consent was obtained from the patient for publication of this case report and any accompanying images. The patient's identity has been protected and no identifiable information is included. A copy of the written consent is available for review by the Editor-in-Chief of this journal on request.

## Funding

This research received no specific grant from any funding agency in the public, commercial, or not-for-profit sectors.

## Declaration of Competing Interest

The authors declare that they have no known competing financial interests or personal relationships that could have appeared to influence the work reported in this paper.
